# Synthesis of Enantiopure Sulfoxides by Concurrent Photocatalytic Oxidation and Biocatalytic Reduction

**DOI:** 10.1002/anie.202117103

**Published:** 2022-03-04

**Authors:** Sarah Bierbaumer, Luca Schmermund, Alexander List, Christoph K. Winkler, Silvia M. Glueck, Wolfgang Kroutil

**Affiliations:** ^1^ Institute of Chemistry, Department of Organic and Bioorganic Chemistry University of Graz NAWI Graz BioTechMed Graz Field of Excellence BioHealth Heinrichstraße 28 8010 Graz Austria

**Keywords:** Biocatalysis, Deracemization, Methionine Sulfoxide Reductases, Photocatalysis, Sulfoxides

## Abstract

The concurrent operation of chemical and biocatalytic reactions in one pot is still a challenging task, and, in particular for chemical photocatalysts, examples besides simple cofactor recycling systems are rare. However, especially due to the complementary chemistry that the two fields of catalysis promote, their combination in one pot has the potential to unlock intriguing, unprecedented overall reactivities. Herein we demonstrate a concurrent biocatalytic reduction and photocatalytic oxidation process. Specifically, the enantioselective biocatalytic sulfoxide reduction using (*S*)‐selective methionine sulfoxide reductases was coupled to an unselective light‐dependent sulfoxidation. Protochlorophyllide was established as a new green photocatalyst for the sulfoxidation. Overall, a cyclic deracemization process to produce nonracemic sulfoxides was achieved and the target compounds were obtained with excellent conversions (up to 91 %) and superb optical purity (>99 % *ee*).

## Graphical abstract

The power of biocatalysis and photocatalysis was combined in one pot for a concurrent reduction–oxidation sequence to gain access to enantiopure sulfoxides. In a cyclic deracemization process the enantioselective biocatalytic reduction is followed by a non‐stereoselective light‐dependent oxidation to yield the desired target compounds in >99 % *ee. rac‐*sulfoxides carrying various substituents on the aromatic ring were well accepted by the system.

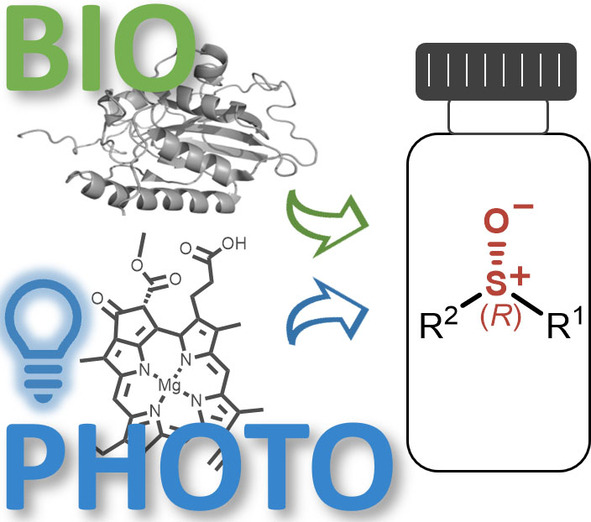

Biocatalysis and photocatalysis constitute two emerging fields with a deep impact on modern synthetic organic chemistry, as they contribute previously inaccessible, sustainable new chemical transformations. The combination of the catalytic power and the excellent selectivities of enzymes in functional group interconversions^[1]^ with the powerful redox‐ and bond‐formation reactions of photo(organo)catalysis, is expected to facilitate the development of novel synthetic strategies.[^2^, ^3^] Furthermore, using light as an environmentally friendly and inexhaustible renewable resource potentially contributes to the greenness of such processes. Photobiocatalytic strategies that have gained considerable attention in the past years include the use of natural photo‐enzymes,^[4]^ and the application of promiscuous reactions of enzymes under illumination,[^5^, ^6^] but also the use of photocatalytic–biocatalytic cascades[^6^, ^7^, ^8^] as well as photocatalytic cofactor recycling systems.^[9]^ However, to unlock their full reactivity, photo‐ and biocatalysts usually require diverging reaction conditions:[^3^, ^4^, ^6^, ^8^, ^10^, ^11^] While enzymatic reactions are best performed in water, most photocatalysts require organic solvents. Furthermore, organocatalytic reactions are often accelerated at high temperatures that are harmful for enzymes. Similarly, photocatalytic reactions are accelerated at higher light intensity and light that is higher in energy, while enzymes (or the associated cofactors) may degrade when they are illuminated with shorter wavelengths. In contrast, applying enzymes in the form of (lyophilized) whole cells or in immobilized form may result in turbid reaction mixtures, resulting in shading and diminished quantum efficiency. Finally, the reactive oxygen species and other reactive (e.g. radical) side products of photocatalytic reactions may be harmful for biocatalysts.[^4^, ^10^] General strategies to overcome these limiting factors include encapsulation of the catalysts,^[12]^ compartmentalization,^[13]^ the use of fine‐tuned reaction media,^[14]^ and most importantly, performing the incompatible reactions sequentially rather than simultaneously.[^15^, ^16^] Besides numerous examples of biocatalytic redox‐reactions that are coupled to a photocatalytic cofactor regeneration,[^4^, ^6^, ^8^] only very few cascades utilizing biocatalytic and photocatalytic transformations are concurrently reported. Examples include the combinations of ruthenium complexes, iridium complexes, carbon nanodots or flavins with alcohol dehydrogenases, ene reductases or transaminases.[^15^, ^17^] In an interesting example an iridium based photocatalyst was combined with a monoamine oxidase for a reductive cyclic deracemization of amines.^[18]^ Recently, also the application of an organo‐photocatalyst and different enzymes in tandem was demonstrated to achieve asymmetric C−H bond functionalizations. However, in this case a separation of the catalysts in space and time was beneficial, underlining the requirement for better compatible photobiocatalytic protocols.^[19]^

In here, we successfully combined the strengths of bio‐ and photocatalysis concurrently in one pot in a cyclic deracemization process to gain access to enantiopure sulfoxides. Due to their biological activities the target compounds are valuable building blocks in various fields of chemical industry (such as to produce APIs, fragrance and flavours).^[20]^ Classic cyclic deracemizations apply stereoselective oxidations together with unselective reductions and after several rounds of oxidation/reduction the less preferred enantiomer remains in high optical purity.^[21]^ The systems regularly suffer from the incompatibility of the parallel oxidation and reduction reactions that are going on in one pot, a problem that is often overcome by utilizing the chemoselectivity of enzymes.^[22]^ A number of concepts for the deracemization of sulfoxides have been reported, including chemical and enzymatic kinetic resolutions,[^23^, ^24^, ^25^] cyclic chemoenzymatic processes,[^26^, ^27^] a photochemical deracemization process^[28]^ and Viedma ripening.^[29]^ Previously described cyclic deracemizations of sulfoxides required one equivalent of an organic oxidant.^[26]^ Interestingly, as an opposite to the classic cyclic deracemizations, using the same process towards chiral sulfoxides requires the combination of a non‐stereoselective oxidation with an asymmetric reduction.

Herein an enantioselective sulfoxide reduction catalyzed by methionine sulfoxide reductases was coupled with an unselective light‐dependent sulfide oxidation, the latter catalyzed by protochlorophyllide, a novel green photocatalyst, to yield the desired optically pure sulfoxides in a rare case of a reductive cyclic deracemization.

To investigate the biocatalytic sulfoxide reduction module, the (*S*)‐selective methionine sulfoxide reductases[^24^, ^25^, ^30^, ^31^] (Msrs) from *Pseudomonas alcaliphila* ^[30]^ (paMsr), *Pseudomonas montelii* ^[24]^ (pmMsr) and *Escherichia coli* ^[31]^ (MsrA) were applied as biocatalysts (Scheme 1, yellow box). Msrs as well as the molybdenum‐containing DMSO reductases^[32]^ (DMSOR) are well described in literature for their ability to perform stereoselective sulfoxide reduction. Msrs are, in contrast to DMSORs generally easily heterologously expressed in *Escherichia coli* (*E. coli*) and show high activities and enantioselectivities as well as a rather wide substrate tolerance. In order to evaluate the stereoselectivity of the enzyme candidates, they were applied in the biocatalytic kinetic resolution of racemic methyl‐*p*‐tolyl sulfoxide (*rac*‐**1 a**), using dithiotreitol (DTT) as reduction equivalent. In all cases exclusively the (*S*)‐sulfoxide was reduced, yielding the corresponding sulfide **1 b** and the remaining (*R*)‐sulfoxide in high enantiomeric excess (*ee* >99 %; see Supporting Information, Figure S4).^1^ The enzyme paMsr was selected for the optimization of the following experiments.

**Scheme 1:**
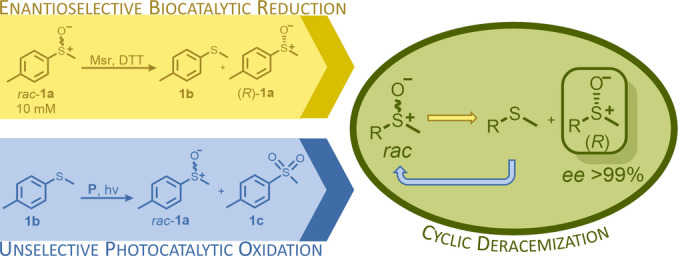
Yellow box (biocatalytic step): kinetic resolution of the model substrate methyl‐*p*‐tolyl sulfoxide (*rac*‐**1 a**) by a (*S*)‐selective methionine sulfoxide reductase (Msr) supplemented with dithiotreitol (DTT) as reduction equivalent; blue box (photocatalytic step): unselective sulfide (**1 b**) oxidation using a photocatalyst (**P**) and light; green circle: cyclic deracemization process by combination of the bio‐ and photocatalytic step yielding optically pure (*R*)‐sulfoxides (*ee*>99 %).

Having a fast and selective biocatalyst for the enantioselective reduction in hand, the unselective oxidation of **1 b** was investigated in greater detail (Scheme 1, blue box). Light‐dependent sulfide oxidation has been reported using a perylene diimide photocatalyst^[33]^ as well as metal catalysts like [Ru(bpy)_3_]^2+^,^[34]^ eosin Y,^[35]^ tetraacetyl riboflavin,^[36]^ porphyrins^[37]^ and thioxanthone derivatives,^[38]^ all almost exclusively applied in organic solvents. Initial experiments were performed, using the commercially available and rather inexpensive 2‐chloro‐thioxanthen‐9‐one **P1** as photocatalyst (**P**) in different solvents (MeOH, EtOH, ACN, H_2_O, see Supporting Information, Table S3). Even though the sulfide oxidation was more efficient in organic solvents (conv. ≈60–70 %), a conversion of around 30 % in water was found, representing a good starting point to run the system in aqueous reaction media. Eight additional photocatalysts besides the above mentioned **P1**, (**P2**‐**P7**) were tested in the oxidation module using the sulfide (**1 b**) as test substrate in aqueous media (KPi buffer; 50 mM, pH 6.5) under illumination at 405 nm. Amongst them protoporphyrin IX (**P2a**), protoporphyrin IX zinc (II) (**P2b**), 5‐(4‐carboxypropylcarbamoylphenyl)‐10,15,20‐(tri‐4‐sulfonatophenyl) porphyrin triammonium (**P3**), 5‐(4‐carboxyphenyl)‐10,15,20‐(triphenyl)porphyrin (**P4**), ruthenium‐tris(2,2′‐bipyridyl) dichloride (**P5**), protochlorophyllide (**P6**) and eosin Y (**P7**). Note that the reaction conditions were slightly altered for **P6** as it is not compatible with the conditions employed for the other catalysts. To our delight, using **P2a**, **P2b** and **P6**, the reaction went to completion (>99 % conv.) giving the corresponding racemic sulfoxide (*rac*‐**1 a**) without any detectable overoxidation to the corresponding sulfone (**1 c**) (Table 1). This demonstrates that the amount of dissolved oxygen in the sealed vials was sufficient for the reaction at this scale. The porphyrin‐based sulfide oxidation is assumed to proceed via a singlet oxygen that is formed in situ, which converts the sulfide substrate into a persulfoxide intermediate, which then symproportionates with a second substrate molecule into two sulfoxides.^[39]^ Eosin Y (**P7**) also performed good, leading to 95 % conversion to the sulfoxide (*rac*‐**1 a**) and a rather low rate of overoxidation (5 % of **1 c**) whereas **P3** – **P5** showed only moderate conversions (62–87 %) going in hand with elevated overoxidation (13–38 %) to **1 c**. Interestingly, when blank reactions under illumination at 385 nm were run in water without the photocatalyst, the model substrate methyl‐*p*‐tolyl sulfide (**1 b**) was oxidized to the corresponding sulfoxide with >99 % conversion whereas running the same blank reaction in MeOH showed no conversion at all (Table 1 and Table S3).^[40]^ Therefore, such conditions were also tested in the next steps.

**Table 1:** Light‐dependent sulfide oxidation catalyzed by various photocatalysts.

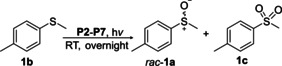				
Photocatalyst	*rac*‐**1 a** [%] **1 c** [%]		Photocatalyst	*rac*‐**1 a** [%] **1 c** [%]
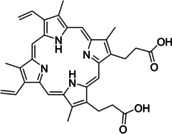	>99 n.d.^[b]^		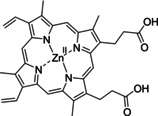	>99 n.d.^[b]^
**P2a**			**P2b**	
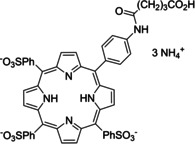	87 13		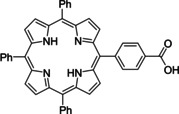	62 38
**P3**			**P4**	
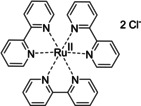	71 29		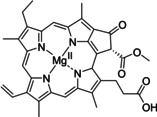	>99 n.d.^[b]^
**P5**			**P6** ^[a,c]^	
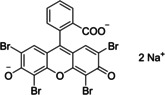	95 5		**no photocatalyst (UV light)** ^[c,d]^	>99 n.d.^[b]^
**P7**

Reaction conditions: photocatalyst (2 mg mL^−1^), **1 b** (20 mM), KPi buffer (50 mM, pH 6.5), 500 μL total reaction volume. Samples were illuminated at 405 nm, 0.99 μmol photons s^−1^, 25 °C, 500 rpm, overnight. For all oxidations recoveries were >80 %. [a] 0.33 mM **P6**, illumination at 455 nm with 0.036 μmol photons s^−1^. [b] n.d.=not detected. [c] 10 mM **1 b** were applied. [d] in H_2_O, illuminated at 385 nm, 0.75 μmol photons s^−1^, 24 h.

Subsequently, the concurrent photocatalyzed oxidation and biocatalytic reduction in one pot was examined. Note that the photon fluxes for the photoreactor were determined by chemical actinometry as previously reported.^[41]^ The five best performing photocatalysts from the preceding oxidation screening were applied in the cyclic deracemization process (Figure 1A). Whereas the use of **P2b**, **P3**, **P5** and **P7** resulted in only moderate *ee*’s for (*R*)‐**1 a** (≈60 %), superb optical purity was obtained with the porphyrin derivative **P6** (*ee* >99 %), which is known as the natural substrate of light‐dependent protochlorophyllide reductases.^[42]^ Note that for cyclic deracemizations the achieved *ee* is a better measure for the reaction than the substrate/product ratio.^[43]^ Testing the spontaneous oxidation at 385 nm revealed also good enantioselectivity and also demonstrated the photostability^[44]^ of the biocatalyst under these conditions (for further details see Supporting Information, Figure S5). Still, as using the photocatalyst **P6** clearly outperformed the other catalysts and also the spontaneous sulfoxidation, it was selected for further optimization. Additionally, applying 385 nm without photocatalyst required a significantly higher DTT concentration and longer wavelengths. It is also worth to mention that protochlorophyllide (**P6**) is a natural compound that was isolated from *Rhodobacter capsulatus* ZY5 cells as previously described (see Supporting Information, section 4) and can therefore be considered a green photoorganocatalyst.^[45]^ **P6** was applied for further optimization studies in order to improve the recovery rate as well as to minimize the amount of remaining intermediate **1 b** in the cyclic deracemization system. Control reactions confirmed that oxidation only occurs in presence of the photocatalyst (see Supporting Information, Table S4). Increasing the concentration of the photocatalyst slightly improved the overall performance of the cyclic system (up to 8 % more enantiopure sulfoxide, see Supporting Information, Figure S7).

**Figure 1:**
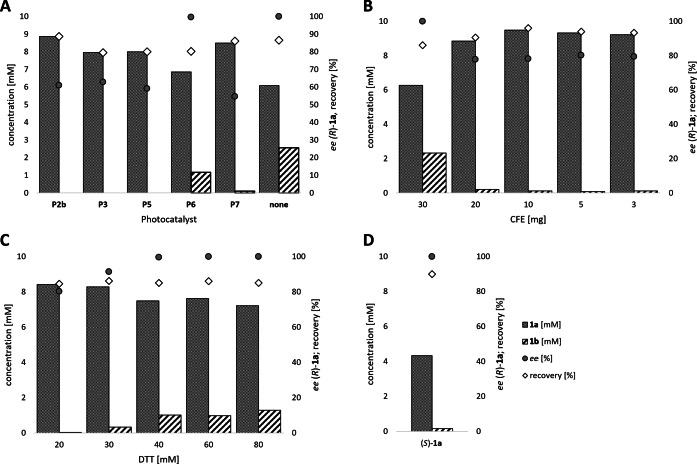
Optimization of the cyclic deracemization process of *rac*‐**1 a**. General reaction conditions: *rac*‐**1 a** (10 mM), DTT (20 mM), paMsr (10 mg CFE), photocatalyst as indicated, KPi buffer (50 mM, pH 7.5), 30 °C, 400 rpm, overnight, total reaction volume: 0.5 mL or as indicated. A) Identification of the best performing photocatalyst in the cyclic deracemization process. Conditions: photocatalyst **P2b**, **P3**, **P5**, **P7** (1 mg) or **P6** (0.33 mM), 2 % v/v MeOH with KPi buffer pH 6.5 or none with paMsr (5 mg CFE) and DTT (50 mM). Illumination: **P2b**/**P3** 405 nm, 0.165 μmol photons s^−1^, **P5** 455 nm, 0.180 μmol photons s^−1^, **P6** 455 nm, 0.036 μmol photons s^−1^, **P7** 528 nm, 0.075 μmol photons s^−1^ or without photocatalyst 385 nm, 0.75 μmol photons s^−1^. B) Optimization of the paMsr CFE concentration in the cyclic deracemization process. Conditions: paMsr CFE (3–30 mg/0.5 mL), **P6** (108 μM), 2 % v/v MeOH, 24 h. C) Optimization of the DTT concentration in the cyclic deracemization process. Conditions: **P6** (108 μM), 2 % v/v MeOH, DTT (20–80 mM), paMsr CFE (3 mg), 20 h. D) Stereoinversion of (*S*)‐**1 a**. Conditions: (*S*)‐**1 a** (5 mM), DTT (50 mM), **P6** (51 μM), 2 % v/v MeOH, paMsr CFE (5 mg), KPi buffer (50 mM, pH 6.0), 24 h.

An evaluation of the optimal enzyme concentration showed that lowering the amount of cell free extract (CFE) resulted in improved recoveries (>90 %) and less intermediate **1 b**, however, also in a decrease of the optical purity of the target sulfoxide (Figure 1B). The lower *ee* may be rationalized by a slower reduction rate or by the fact that less reduction equivalents are supplied in form of the applied CFE. A control reaction confirmed, that 30 mg of paMsr CFE entirely resolved 10 mM racemic sulfoxide without the addition of external reducing agent (DTT), while 10 mg paMsr CFE reduced less than 1 mM of *rac*‐**1 a** (see Supporting Information, Figure S8).

Due to the low stability of DTT an excess of the reducing agent is needed.^[46]^ A variation of the supplied DTT amount showed, that above 40 mM DTT, enantiopure **1 a** is produced, but also 12–15 % of the intermediate **1 b** is obtained (Figure 1C). A time study (see Supporting Information, Figure S9) revealed that the oxidation slowed down after 5 h, which explains the increased amounts of the sulfide intermediate (**1 b**) at the reaction's endpoint. Investigation of the optimal reaction pH revealed that pH‐adjustment offers a simple tool to balance the rate of oxidation and reduction, as lower pH‐values (pH 5.8) support the oxidation going in hand with less sulfide intermediate (**1 b**) while the paMsr is still active (see Supporting Information, Table S6).

As additional proof for the deracemization, a stereoinversion starting from the enantiopure (*S*)‐sulfoxide [(*S*)‐**1 a**] was performed yielding solely the mirror image (*R*)‐sulfoxide [(*R*)‐**1 a**] (Figure 1D).

Finally, all investigated methionine sulfoxide reductases were applied in the cyclic deracemization process under optimized reaction conditions. In all cases successful sulfoxide deracemization was achieved, yielding optically pure (*R*)‐sulfoxide [(*R*)‐**1 a**] and only trace of the corresponding sulfide intermediate (**1 b**, 2–4 %) (Table 2). Similar results were obtained when purified paMsr was used which, however, required the addition of triton X‐100 as additive, most likely to inhibit the formation of **P6**‐aggregates in water (see Supporting Information, Figure S10).^[47]^

**Table 2:** Deracemization of *rac*‐**1 a** under optimized reaction condition using various Msrs.^[a]^

Entry	Substrate	[mM]	Biocatalyst	*ee* of (*R*)‐**1 a** [%]	**1 b** [%]
1	*rac*‐**1 a**	10	paMsr	>99	2
2	*rac*‐**1 a**	10	pmMsr	>99	4
3	*rac*‐**1 a**	10	MsrA	>99	4

[a] Reaction conditions: *rac*‐**1 a** (10 mM), DTT (50 mM), KPi buffer (50 mM, pH 6.0), 2 % v/v MeOH, **P6** (51 μM), biocatalyst (5 mg, CFE), 0.5 mL final reaction volume, 30 °C, 400 rpm, 455 nm, 0.178 μmol photons s^−1^, 24 h; recoveries were >90 %.

To demonstrate the applicability of the established reductive cyclic deracemization, a range of structurally and electronically diverse *rac*‐sulfoxide substrates were tested. Substrates bearing electron‐donating (**3 a**, **4 a**, **6 a**) as well substrates with as electron‐withdrawing substituents (**8 a**), either in the *meta*‐ or *para*‐position of the aromatic ring, were deracemized with recoveries of 59–91 % and with up to >99 % *ee* (Figure 2, **2 a**–**6 a**, **8 a**). Substrate **7 a**, bearing a benzylic substituent was also converted with high selectivity and almost quantitative recovery. While limited stereoselectivity has been reported for paMsr for sulfoxides bearing two small alkyl groups,^[30]^ the long‐chain sulfoxide **9 a** was deracemized to enantiopurity. Finally, the system was applied for the deracemization of the anti‐inflammatory prodrug sulindac (**10 a**). While **10 a** is marketed as racemate, its (*R*)‐enantiomer was proven to be utilized more efficiently.^[48]^ Due to the compound's instability under illumination, the reaction time and the illumination intensity were reduced leading still to an excellent optical purity of >99 % *ee*, albeit at reduced recovery of (*R*)‐**10 a** (43 %). Note that this yield can also be reached in a classic kinetic resolution.

**Figure 2:**
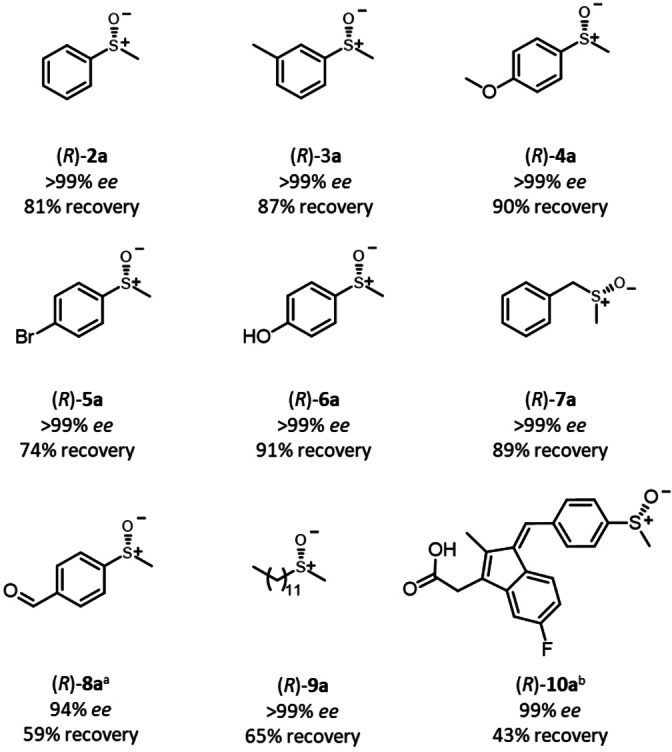
Substrate scope of the cyclic deracemization. Reaction conditions: *rac*‐**2 a**–**10 a** (10 mM), paMsr (5 mg CFE), DTT (50 mM), 2 % v/v MeOH, **P6** (51 μM), KPi buffer (50 mM, pH 6.0), 0.5 mL final reaction volume, 30 °C, 400 rpm, 455 nm, 0.036 μmol photons s^−1^, 24 h. Recovery and *ee* determined via HPLC measurements (see Supporting Information, section 7, 8 and 10). [a] Purified protein was applied to avoid side reactions. Reaction time: 6 h. [b] KPi buffer (100 mM, pH 8.0, final pH of reaction mixture 7.5), 0.018 μmol photons s^−1^, reaction time: 5 min.

In summary, we successfully combined the power of photo‐ and biocatalysis in one pot to establish a concurrent reduction–oxidation sequence to perform a reductive cyclic deracemization, gaining access to optically pure sulfoxides. The target molecules represent widespread structural motives of numerous biologically active compounds. The concept was further optimized to demonstrate the complete stereoinversion of the (*S*)‐enantiomer of the model sulfoxide substrate [(*S*)‐**1 a**] to the corresponding mirror image (*R*)‐**1 a**. Furthermore, the generality of the system was demonstrated by applying various biocatalysts as well as structurally diverse substrates to yield the desired sulfoxides in absolute optical purity. The utilization of an enzyme and light as catalytic power under ambient reaction conditions complies with the requirement for more sustainable and environment‐friendly synthetic strategies in modern chemical production. Finally, we established protochlorophyllide, isolated from a *Rhodobacter* strain as a novel green photocatalyst, contributing to the sustainability of the process.

## Conflict of interest

The authors declare no conflict of interest.

## Supporting information

As a service to our authors and readers, this journal provides supporting information supplied by the authors. Such materials are peer reviewed and may be re‐organized for online delivery, but are not copy‐edited or typeset. Technical support issues arising from supporting information (other than missing files) should be addressed to the authors.

Supporting Information

## Data Availability

The data that support the findings of this study are available in the supplementary material of this article.

## Footnotes

n1 Due to incomplete recoveries, no E‐values were calculated. Note that one of the requirements for the calculation of an accurate E‐value is, that no side reactions are going on.
